# Emerging Strategies for Aflatoxin Resistance in Peanuts via Precision Breeding

**DOI:** 10.3390/toxins17080394

**Published:** 2025-08-06

**Authors:** Archana Khadgi, Saikrisha Lekkala, Pankaj K. Verma, Naveen Puppala, Madhusudhana R. Janga

**Affiliations:** 1 Institute of Genomics for Crop Abiotic Stress Tolerance (IGCAST), Department of Plant and Soil Sciences, Texas Tech University, Lubbock, TX 79403, USA; 2 Department of Plant and Environmental Sciences, New Mexico State University, Clovis, NM 88101, USA

**Keywords:** aflatoxin, *Aspergillus flavus*, susceptibility genes, *Arachis hypogaea*, CRISPR/Cas9 genome editing, host-induced gene silencing, GWAS, QTL, breeding, MAS

## Abstract

Aflatoxin contamination, primarily caused by *Aspergillus flavus*, poses a significant threat to peanut (*Arachis hypogaea* L.) production, food safety, and global trade. Despite extensive efforts, breeding for durable resistance remains difficult due to the polygenic and environmentally sensitive nature of resistance. Although germplasm such as J11 have shown partial resistance, none of the identified lines demonstrated stable or comprehensive protection across diverse environments. Resistance involves physical barriers, biochemical defenses, and suppression of toxin biosynthesis. However, these traits typically exhibit modest effects and are strongly influenced by genotype–environment interactions. A paradigm shift is underway with increasing focus on host susceptibility (S) genes, native peanut genes exploited by *A. flavus* to facilitate colonization or toxin production. Recent studies have identified promising S gene candidates such as *AhS5H1/2*, which suppress salicylic acid-mediated defense, and *ABR1*, a negative regulator of ABA signaling. Disrupting such genes through gene editing holds potential for broad-spectrum resistance. To advance resistance breeding, an integrated pipeline is essential. This includes phenotyping diverse germplasm under stress conditions, mapping resistance loci using QTL and GWAS, and applying multi-omics platforms to identify candidate genes. Functional validation using CRISPR/Cas9, Cas12a, base editors, and prime editing allows precise gene targeting. Validated genes can be introgressed into elite lines through breeding by marker-assisted and genomic selection, accelerating the breeding of aflatoxin-resistant peanut varieties. This review highlights recent advances in peanut aflatoxin resistance research, emphasizing susceptibility gene targeting and genome editing. Integrating conventional breeding with multi-omics and precision biotechnology offers a promising path toward developing aflatoxin-free peanut cultivars.

## 1. Introduction

*Aspergillus flavus* is a fungal pathogen and the primary source of aflatoxin contamination in a wide range of crops, posing a constant threat to food safety and crop productivity worldwide [1]. Due to its lack of host specificity, *A. flavus* infects numerous plant species, but its impact is particularly severe in peanuts [2,3,4,5,6]. The fungus thrives in hot climates and becomes especially problematic in regions where peanuts are exposed to high temperatures, drought, and insect damage. These stressors not only weaken plant defenses but also create physical entry points that facilitate fungal invasion and aflatoxin production [7,8,9,10]. Following entry through wounds, *A. flavus* can penetrate the shell and seed coat and establish within the cotyledon, often resulting in reduced germination and seedling vigor [11]. The pathogen persists in infected plant debris such as conidia, sclerotia, or mycelia, enabling survival between growing seasons [12]. Under favorable conditions, sclerotia start germinating and lead to the production of mycelia and conidiophores [13,14]. These structures generate conidia, which can spread via air currents, water splashes, or insect vectors. Insects such as *Helicoverpa armigera* and *Caryedon serratus* play a significant role in dispersal and infection by creating entry points in plant tissues or directly transporting spores [15].

Ultimately, *A. flavus* infection leads to the accumulation of aflatoxins, highly toxic, carcinogenic secondary metabolites that pose serious health and trade risks. Aflatoxins are primarily produced by *A. flavus* and *A. parasiticus*, with key analogs including AFB_1_, AFB_2_, AFG_1_, AFG_2_, AFM_1_, and AFM_2_. Among these, AFB_1_ is the most prevalent and toxic in peanuts. It is a potent hepatocarcinogen that forms DNA adducts in the liver, contributing to mutations in tumor suppressor genes such as *TP53* [16]. Chronic exposure in children has been linked to stunted growth, micronutrient deficiencies, and immune suppression [17,18]. In animals, ingestion of contaminated peanut meal reduces productivity and can result in the transfer of AFM_1_ into milk and dairy products [19,20].

Although improved irrigation, proper drying, and secure storage can reduce aflatoxin contamination to some extent, these measures alone are insufficient to fully address the problem. Genetic resistance remains the most cost-effective and sustainable long-term solution. Resistance to *A. flavus* infection and aflatoxin accumulation in peanut is now recognized as a complex, multigenic trait that is strongly influenced by environmental factors [10]. However, progress in breeding aflatoxin-resistant cultivars has been limited due to low levels of resistance in the available germplasm and the complex, multigenic nature of the resistance mechanisms [21]. Recent breeding strategies are shifting focus from the identification of major resistance (R) genes to targeting host susceptibility (S) genes. Disruption of such S genes can provide broad-spectrum, and durable resistance without the need for transgenic approaches. Advances in transcriptomics and proteomics have enabled the identification of hundreds of differentially expressed genes between resistant and susceptible peanut genotypes, many of which are involved in defense signaling, oxidative stress response, and producing secondary metabolites [22,23,24,25]. These findings provide a valuable foundation for pinpointing candidate S genes and deciphering the underlying molecular networks that govern resistance.

This review centers on peanut resistance mechanisms to *A. flavus* and reduced aflatoxin contamination, with an emphasis on recent insights into host susceptibility genes and pathways that could be manipulated to enhance resistance. We discuss advances in conventional breeding and QTL mapping, summarize new biotechnological approaches like host-induced gene silencing, and highlight emerging evidence for peanut S genes that could be targeted by modern gene editing tools to confer aflatoxin resistance. By integrating these diverse strategies, we aim to outline a path forward for developing peanut cultivars with enhanced and durable resistance to aflatoxin contamination.

## 2. Genetic and Molecular Regulation of Aflatoxin Biosynthesis

Producing aflatoxins in *A. flavus* is a developmentally coordinated and tightly regulated process governed by a complex network of genetic and environmental signals. The availability of its complete genome sequence, along with a comprehensive microarray platform, has accelerated insights into the molecular mechanisms driving aflatoxin biosynthesis [26].

### 2.1. Aflatoxin Biosynthesis

**Figure 1:**
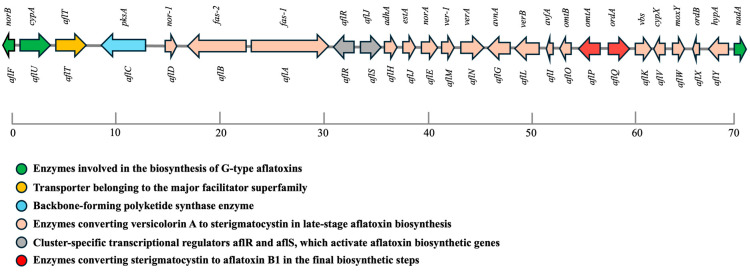
Genomic organization of the aflatoxin biosynthetic gene cluster in *A. flavus*. The ~70 kb gene cluster contains structural, regulatory, transporter, and tailoring genes required for aflatoxin biosynthesis. Polyketide synthesis is initiated by *aflC* (pksA), followed by sequential action of enzymes synthesizing sterigmatocystin and other intermediates (peach), leading to B- and G-type aflatoxins. Green arrows indicate genes specific to G-type aflatoxin biosynthesis; red arrows represent enzymes finalizing aflatoxin formation. Major facilitator transporter genes (*aflT*) are highlighted in yellow, and regulatory elements (*aflR*, *aflS*) in gray. The spatial clustering of these genes underpins coordinated pathway activation during infection.

Aflatoxins are synthesized via a highly coordinated polyketide-derived biosynthetic pathway involving over two dozen enzymatic transformations [2,26,27,28,29]. The genes responsible for aflatoxin biosynthesis are clustered within a ~70-kilobase region near the telomeric end of chromosome 3 (Figure 1). This cluster includes about 30 co-regulated genes, designated with the prefix “*afl*”, ranging from *aflA* to *aflY* [2,26,30]. These genes encode enzymes responsible for specific transformations in the biosynthetic pathway. The pathway begins with the polymerization of acetate units into a polyketide backbone, followed by successive steps that yield intermediates such as norsolorinic acid (NOR), averantin (AVN), 5′-hydroxyaverantin (HAVN), oxoaverantin (OAVN), averufin (AVF), hydroxyversicolorone (HVN), versiconal hemiacetal acetate (VHA), versiconol acetate (VOAc), versiconal (VOH), versicolorin B (VERB), versicolorin A (VERA), demethylsterigmatocystin (DMST), sterigmatocystin (ST), O-methylsterigmatocystin (OMST), and 11-hydroxy-OMST, before culminating in AFB_1_ [28].

### 2.2. Transcriptional Regulation of Aflatoxins Biosynthetic Genes, aflR and aflS

Transcription of the aflatoxin cluster is mainly controlled by two key regulators: *aflR* and *aflS*. These genes are adjacent to one another in the gene cluster and are transcribed divergently from closely spaced promoters [29,31]. *aflR* encodes a zinc finger DNA-binding protein that serves as the principal transcriptional activator of the pathway. Its overexpression leads to a 50-fold increase in aflatoxin production, whereas its deletion nearly abolishes toxin biosynthesis [32,33]. Moreover, chemical inhibitors of aflatoxin production often exert their effects by downregulating *aflR* expression [34,35]. *aflS* plays a supporting role by enhancing *aflR*-mediated activation of biosynthetic genes. Overexpression of *aflS* in *A. flavus* has been shown to increase aflatoxin levels further [29,36], and in *A. parasiticus*, evidence suggests a physical interaction between *aflS* and *aflR*, potentially enhancing *aflR*’s promoter binding efficiency [31].

### 2.3. Developmental, Epigenetic, and Environmental Regulation of Aflatoxin Biosynthesis

Aflatoxin biosynthesis is intricately linked with fungal development and morphogenesis. Genes regulating conidiophore and sclerotial development also impact toxin production. For example, *hbx1* deletion completely halts aflatoxin production and developmental differentiation, in part through downregulation of cluster genes including *aflC*, *aflD*, *aflM*, and *aflR* [37]. Additional transcriptional regulators such as *NsdC* and *NsdD* are essential for sclerotia formation and mycotoxin biosynthesis [38,39]. Other important factors include *AfRafA*, *AfStuA*, *AflRsmA*, *AflSkn7*, and *Rum1*, all of which modulate developmentally associated secondary metabolism [40,41,42,43,44]. Furthermore, disruption of genes such as *hexA*, *sakA*, *aflPex5*, and *Aflndk* has been shown to affect sporulation, stress response, and aflatoxin yield [43,45,46].

A central component of this regulatory network is the Velvet complex, comprising VeA and LaeA. This complex is a hub that integrates light signaling, developmental control, and secondary metabolism. Deletion of either *veA* or *laeA* leads to complete loss of both aflatoxin production and sclerotium formation [47,48,49]. Genes involved in hyphal fusion, such as *hamF*, *hamG*, *hamH*, and *hamI*, also play a role, with their deletion resulting in decreased aflatoxin production and developmental defects [50]. The kinetochore-associated protein Spc105, which physically interacts with LaeA, is also necessary for proper conidiophore and sclerotial development [51].

Beyond intrinsic genetic control, aflatoxin biosynthesis is strongly influenced by environmental cues. Factors such as temperature, pH, oxidative stress, and nutrient availability can activate signaling cascades that modulate the expression of aflatoxin biosynthetic genes [6,28]. These environmental stimuli not only affect growth and development but also integrate with global regulators such as *LaeA* and *aflR* to fine-tune the metabolic output of the fungus. A comprehensive understanding of these interactions is crucial for devising effective strategies to mitigate aflatoxin contamination in crops under variable field and storage conditions. Understanding the fungal genes that drive aflatoxin production also identifies molecular targets for interventions; for instance, host-induced gene silencing can target some of these fungal regulators. We next examine how peanuts defend themselves and how we can leverage both peanut and fungal genes for resistance.

## 3. Structural, Biochemical, and Induced Defenses in Peanut Against *A. flavus* Infection

**Figure 2:**
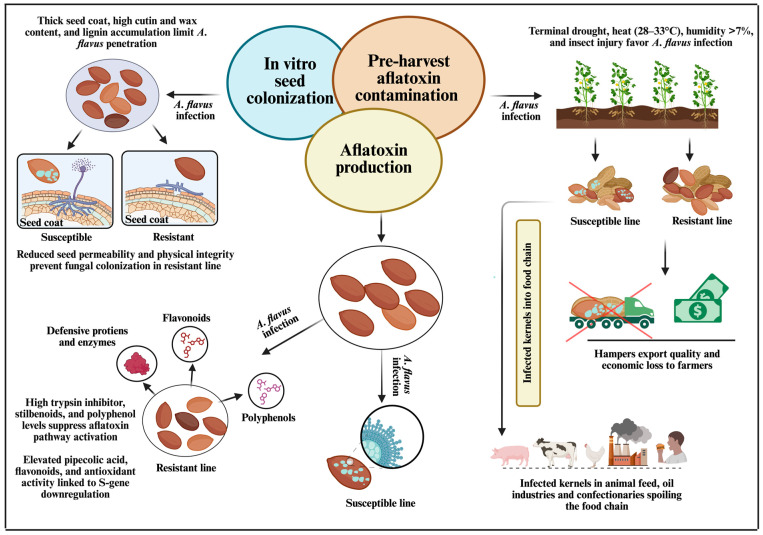
Multifaceted resistance mechanism in peanut against *A. flavus,* typically classified into three main categories: resistance to seed colonization (IVSC), resistance to pre-harvest aflatoxin contamination (PAC), and resistance to aflatoxin production (AP).

Understanding the natural resistance mechanisms of peanut against *A. flavus* infection and subsequent aflatoxin contamination has been a major focus of research for decades. This resistance is multifaceted, involving structural barriers, biochemical pathways, and inducible defense responses that operate at different stages of the infection process. Resistance against *A. flavus* is typically classified into three main categories including resistance to seed colonization (IVSC) (the ability of seeds to restrict fungal growth), resistance to pre-harvest aflatoxin contamination (PAC) (limiting toxin accumulation in the field), and resistance to aflatoxin production (AP) (suppressing toxin biosynthesis even after infection) [21,24,52] (Figure 2). The genetic basis of resistance to *A. flavus* in peanut is complex and predominantly quantitative, involving the interplay of multiple genes [9]. This polygenic nature, coupled with strong gene-environment (G × E) interactions, poses significant challenges for breeding programs. The expression and effectiveness of resistance traits can vary widely among peanut genotypes and are often inconsistent across different environmental conditions [10,53]. Even when additive genetic effects are present, environmental variability can limit the stability and utility of resistant varieties, complicating their deployment in diverse agroecological zones [7,10].

### 3.1. Preformed Structural Barriers, Pod Shell, and Seed Coat Serve as Physical Barriers for A. flavus Infection

Peanuts employ robust structural defenses to mitigate *A. flavus* infection, with the pod shell and seed coat forming the first line of defense. The shell’s architecture, particularly its thickness, texture, and permeability, has been linked to reduced infection rates, especially in resistant cultivars [7,53,54]. These structural traits act as mechanical barriers that restrict fungal entry. Similarly, the seed coat, the outermost layer of the peanut kernel, serves a dual role as a physical and chemical barrier. Its resistance potential has been associated with morphological features such as thickness, density, and biochemical content [11,53]. These features, when absent or underdeveloped, may represent structural susceptibility factors that facilitate infection.

### 3.2. Biochemical Defenses—Lignin, Waxes, Phenolics, and Flavonoids

Specialized compounds in the pod and seed coat offer further protection. Lignin, abundant in wild peanut species, contributes to cell wall rigidity and resistance to mechanical and biological stress [55]. Earlier LaPrade (1973) proposed that resistance may correlate with seed coat thickness and structural complexity [56]. Wax and cutin layers add hydrophobicity, minimizing water retention and fungal adhesion, thereby acting as chemical barriers against *A. flavus* [57]. In addition to structural defenses, peanut tissues are enriched with antifungal phytochemicals. Phenolic compounds such as *p*-coumaric acid, ferulic acid, hydroxybenzoic acid, and chlorogenic acid have been identified in the seed coat, along with flavonoids like epicatechin, quercetin, and resveratrol [58,59]. These metabolites possess antioxidants and antimicrobial activity and are believed to contribute significantly to fungal resistance [60,61,62]. Among the most potent biochemical defenses are secondary metabolites and enzymatic inhibitors that directly interfere with fungal physiology. Tannins in the peanut testa have also been reported to inhibit *A. flavus* growth [11,63]. Likewise, specific antifungal molecules such as trypsin inhibitors and 5,7-dimethoxy isoflavone have been linked to enhanced resistance in certain peanut lines [64,65]. More recently, Sharma et al. (2021) identified pipecolic acid (Pip) as a resistance-associated metabolite, showing higher accumulation in *A. flavus*-challenged resistant genotypes, highlighting its emerging role in plant immunity [66]. Recent complementary profiling across drought-stressed and well-watered lines (e.g., Zhonghua 6 vs. Yuanza 9102) identified elevated resveratrol, cinnamic, coumaric and ferulic acids, and 13S-HPODE in resistant seeds, all correlating with reduced fungal colonization [67]. A recent study profiled peanut seed coats and identified phenylpropanoid-derived metabolites with antifungal activity, notably 2,5-dihydroxybenzaldehyde, which inhibited *A. flavus* growth by 98.7% and nearly eliminated aflatoxin contamination in vitro [11]. These findings highlight how enhanced metabolite synthesis in resistant varieties can mitigate fungal invasion and mycotoxin accumulation. Deficiency in such compounds may therefore be a biochemical susceptibility trait influencing breeding outcomes.

### 3.3. Induced Defense by Signaling—PTI, ETI, and MAPK Pathways

When *A. flavus* infects peanut plants, the host mounts an innate immune response composed of two interconnected layers: PAMP-triggered immunity (PTI) and effector-triggered immunity (ETI). PTI begins with the recognition of pathogen-associated molecular patterns (PAMPs) by pattern recognition receptors (PRRs) at the plant cell surface, which activate early defense responses such as reactive oxygen species (ROS) bursts, mitogen-activated protein kinase (MAPK) cascades, and transcription of resistance genes. In contrast, ETI is initiated by the direct recognition of pathogen effectors via intracellular nucleotide-binding site leucine-rich repeat (NBS-LRR) proteins, often leading to hypersensitive response (HR) and localized cell death [68]. Recent transcriptomic analyses have revealed that these immune responses are robust in certain peanut genotypes. Cui et al. (2022) reported significant upregulation of MAP kinases (*Arahy.L410JY*, *Arahy.BC5GM2*), cytochrome P450, and the PRR gene RPVOD7 as part of the PTI response [69]. Simultaneously, six NBS-LRR genes and a serine/threonine kinase (*Arahy.D2YYPY*) were highly expressed in resistant cultivars during *A. flavus* infection, indicating a coordinated PTI-ETI response [69,70]. These differences suggest immunological signaling loci represent molecular susceptibility checkpoints worth targeting.

### 3.4. Hormonal Crosstalk and Lipoxygenase (LOX) Pathway

The interplay between jasmonic acid (JA), salicylic acid (SA), and ethylene forms the core of the peanut’s hormonal defense network. These signaling molecules regulate key resistance genes and coordinate responses against biotic stress. This complex hormonal orchestration is fine-tuned by transcription factor (TF) families like WRKY, bZIP, ERF, MYB, MYC, and NAC, which modulate downstream defense mechanisms such as ROS production, synthesis of antifungal secondary metabolites, and expression of pathogenesis-related (PR) proteins [23,24,57,71,72]. Moreover, oxylipins, which are lipid-derived signals, add another regulatory layer. Fungal oxylipins enhance fungal colonization, while plant-derived oxylipins act antagonistically to suppress aflatoxin biosynthesis in *A. flavus* [73]. Peanut plants challenged by *A. flavus* infection, especially under drought stress, exhibit a pronounced oxidative burst marked by the accumulation of ROS. This response is intricately linked to increased lipoxygenase (LOX) pathway activity. LOX enzymes catalyze lipid peroxidation and generate signaling molecules that reinforce cell walls, promote phytoalexin biosynthesis, and contribute to hypersensitive cell death [74]. Their activity is also associated with producing defense-related fatty acids, including JA and methyl-JA, which further amplify immune responses [72,75,76]. LOX pathway deficiencies may therefore identify hormonal susceptibility points.

Notably, the SA and JA signaling pathways often act antagonistically, with activation of one typically suppressing the other [77,78]. SA-mediated defenses are more effective against biotrophic pathogens, whereas JA signaling is central to resistance against necrotrophs. *A. flavus*, a predominantly saprophytic soil fungus, can become parasitic by opportunistically invading developing seeds or senescent, stressed tissues, particularly under environmental stress such as drought or high temperature [79,80]. Consequently, JA is the dominant hormone mediating resistance to aflatoxin-producing fungi. Peanut genotypes with resistance to *A. flavus* show stronger induction of JA-responsive genes and LOX activity, while susceptible lines tend to display elevated SA signaling [24]. Similar trends have been observed in maize, where disruption of JA biosynthesis (e.g., ZmLOX3 knockouts) increased susceptibility to *Aspergillus* spp. and aflatoxin contamination [81].

### 3.5. Pathogenesis-Related Proteins and Phytoalexin Accumulation

Pathogenesis-related proteins and phytoalexins form a critical layer of the peanut’s defense system. Resistant cultivars accumulate higher levels of phenylalanine ammonia-lyase (PAL), which is pivotal for lignin and phytoalexin biosynthesis, and glutathione S-transferases (GSTs), which detoxify ROS and support stress resilience [82]. Enzymes like chitinases and β-1,3-glucanases are also more abundant in resistant lines such as GT-YY9 and GT-YY20, where they degrade fungal cell walls and release PAMPs, thereby enhancing immunity [83,84]. Among the phytoalexins, resveratrol has been documented for its antifungal activity, effectively inhibiting *A. flavus* spore germination and hyphal growth [85]. However, drought stress can compromise this defense by suppressing phytoalexin production [86]. Additional flavonoids, such as quercetin, are also involved in host defense, as they have been shown to downregulate aflatoxin biosynthesis genes in the pathogen [87]. Failure to induce effective chemical defenses may define chemical susceptibility, a critical target for breeding and editing efforts.

## 4. Genetic and Genomic Strategies for Enhancing Aflatoxin Resistance in Peanut

Breeding for resistance against *A. flavus* infection is considered a cost-effective and sustainable approach to mitigate aflatoxin contamination [72]. However, efforts have been hindered by the narrow genetic base of cultivated peanut, the complex, quantitative nature of resistance, which is heavily influenced by environmental interactions, and the lack of elite parental lines that consistently exhibit strong and stable resistance to infection [88,89]. Despite these challenges, screening of diverse germplasm has led to identifying resistant genotypes [9]. Assessing resistance typically involves a combination of phenotypic evaluations, such as the percent seed infection index, visual scoring of disease symptoms, and aflatoxin quantification, and molecular tools like QTL mapping and marker-assisted selection [90]. These methods, alongside functional genomics, have collectively expanded our understanding of the genes and pathways that govern peanut and *A. flavus* interactions.

### 4.1. Genomic Mapping of Resistance Loci Against A. flavus in Peanut

Quantitative trait loci (QTL) studies have identified several genomic regions associated with resistance traits. Liang et al. (2009) mapped six QTLs using three recombinant inbred line (RIL) populations, identifying key regions on chromosomes A01, A02, and B05, each explaining over 10% of phenotypic variation [91]. Similarly, Yu et al. (2019) detected 14 QTLs in a Zhonghua 10 × ICG 12,625 population, including qPSIIA10, which consistently explained 11.32–13% PVE [90]. For aflatoxin levels, overlapping QTLs qAFB1A07, qAFB1B06.1, qAFB2A07, and qAFB2B06 were reported, with individual PVE values reaching up to 21.02%. More recent studies continue to uncover stable and environment-specific QTLs. For example, Khan et al. (2020) reported qRAF-3-1 and qRAF-14-1 on chromosomes A03 and B04 [92], respectively, Jiang et al. (2021) identified six QTLs in a Zhonghua 16 × J11 population, with qPSIIB10 demonstrating consistency across four years [93]. Jin et al. (2023) found additional QTLs (qAFTB05.2, qAFTA05.1, qAFTB06.3) and used conditional mapping to reveal additive effects [94], while Yu et al. (2024) highlighted qAFTsA07.1, which consistently accounted for 13.39% PVE [95].

### 4.2. Multi-Omics Insights into Resistance Mechanisms

Functional genomics has complemented QTL studies by identifying differentially expressed genes (DEGs), proteins (DEPs), and metabolites linked to resistance. Wang et al. (2010) reported 12 DEPs between resistant YJ-1 and susceptible Yueyou 7 genotypes [96], and Guo et al. (2011) identified 62 upregulated genes, including lipoxygenase and PR10, using microarrays [22]. Wang et al. (2012) expanded the analysis by identifying 490 resistance-associated genes using a custom microarray, including 64 defense-related genes [25], while Nayak et al. (2017) and Korani et al. (2018) [23,24] found 4445 and 4272 DEGs in J11 vs. JL24 and ICG 1471 vs. Florida-07, respectively. Zhao et al. (2019) detected 663 DEGs and 314 DEPs in J11, further solidifying its resistance profile [97]. Additional layers of insight were provided by recent multi-omics studies revealing complex regulation at transcript, protein, and metabolite levels [67,69,98]. Across these datasets, several families of defense-related genes consistently appear. These include oxidative stress regulators such as glutathione S-transferases (GSTs), transcription factors like WRKY and MYB, and enzymes associated with pathogenesis-related (PR) responses, late embryogenesis abundant (LEA) proteins, defensins, and lipoxygenases, all of which are upregulated in resistant lines, indicating their central role in defense.

### 4.3. GWAS and SNP Marker Discovery

Genome-wide association studies (GWAS) have complemented QTL and transcriptomic approaches by uncovering specific single-nucleotide polymorphisms (SNPs) associated with resistance traits. Yu et al. (2020) performed GWAS on 99 accessions, identifying 60 SNPs linked to aflatoxin resistance, explaining 16.87–31.70% PVE [89]. Two resistant accessions, Zh.h0551 and Zh.h2150, were particularly noteworthy. Marker peaks were spread across 11 chromosomes, highlighting the polygenic nature of resistance. Ding et al. (2022) identified 16 SNPs associated with shell and seed infection indices (SLII, SDII) and aflatoxin content [99]. Candidate genes near these loci included MYB transcription factors such as *Arahy.J7VJ5I* and NLR-type resistance genes like *Arahy.R1ATPI*, suggesting a role for transcriptional regulation and innate immunity in peanut resistance.

### 4.4. Case Study—The J11 Genotype

The Indian peanut cultivar J11 has emerged as a model genotype for resistance to *A. flavus*. Known for its early maturity, drought tolerance, and seed coat-related traits, J11 has shown strong resistance since the 1980s, first identified through in vitro seed colonization (IVSC) assays. Transcriptomic and proteomic studies have since supported this resistance. Zhao et al. (2019) found 663 DEGs and 314 DEPs post-infection in J11 [97], while Nayak et al. (2017) reported 4445 DEGs [24]. QTL mapping studies by recent studies further demonstrated that J11 contributes major alleles for both infection resistance and aflatoxin suppression [93,94]. Its consistent performance makes J11 an invaluable resource for resistance breeding and gene discovery.

### 4.5. Integrating Omics for Improving Resistance

The combined application of QTL mapping, GWAS, transcriptomics, and proteomics highlights the complexity of peanut resistance to *A. flavus*. Resistance is clearly polygenic and modulated by both genetic background and environmental conditions. The consistent identification of stress-mitigation enzymes, transcription factors, and PR proteins in resistant genotypes reinforces their relevance as breeding targets. These insights support the development of marker-assisted selection and genomic prediction models and open avenues for precise genome editing to enhance aflatoxin resistance in elite peanut cultivars. While most conventional breeding efforts have concentrated on accumulating favorable resistance alleles from diverse germplasm, there is growing recognition that enhancing resistance durability may also require eliminating or modifying the plant’s own susceptibility factors. These susceptibility genes host loci that pathogens exploit to promote colonization or toxin production and represent promising new targets for genetic intervention. Genomic tools now enable not only the stacking of resistance QTLs, but also the precise knockout or suppression of S genes such as *MLO*, *AhS5H1*, and *ABR1* using CRISPR-based editing. These genes, when inactivated, may confer broad-spectrum and stable resistance by disrupting pathogen compatibility at its source. The following sections explore these innovative strategies that bridge classical resistance breeding with targeted genome engineering to combat aflatoxin contamination more effectively [100,101,102].

## 5. Transgenic Strategies—Silencing Pathogen Genes and Boosting Host Defenses

Innovative gene silencing and overexpression approaches have emerged as powerful tools to enhance peanut resistance to *A. flavus* infection and suppress aflatoxin accumulation. Host-induced gene silencing (HIGS) and RNA interference (RNAi) have shown notable promise. RNA interference (RNAi) operates within the plant to silence its own genes via small interfering RNAs (siRNAs), while host-induced gene silencing (HIGS) is a transgenic approach that enables plants to produce dsRNAs targeting pathogenic genes, thereby triggering cross-kingdom gene silencing to suppress infection [103,104,105]. In a foundational study, Sharma et al. (2018) employed HIGS to target two crucial aflatoxin biosynthesis genes, *aflM* and *aflP*, substantially reducing aflatoxin levels [106]. Complementarily, the overexpression of antifungal defensins *MsDef1* and *MtDef4.2* in the susceptible peanut variety JL 24 significantly limited fungal colonization and toxin production. Strikingly, combining these two strategies, HIGS for metabolic suppression and defensin overexpression for fungal resistance, produced peanut lines with robust, cross-morphotype resistance, often achieving aflatoxin levels as low as 1–2 ppb or even undetectable in some events. Building on this success, Prasad et al. (2023) implemented a multiplexed HIGS system targeting multiple developmental and biosynthetic genes *nsdC*, *veA*, *aflM*, and *aflR* [107]. These transgenic peanut lines exhibited significantly reduced fungal colonization and aflatoxin biosynthesis. The proteomic analysis confirmed the suppression of fungal proteins central to toxin production and morphogenesis, including VelC, AflC, AflL, AflM, AflQ, AflR, AflS, AflV, AflW, VeA, and AflJ [107].

### 5.1. Targeting aflR, aflM, veA, and Other Key Regulators

RNAi strategies have further demonstrated success in silencing essential genes in the aflatoxin pathway. Two different studies effectively reduced aflatoxin accumulation by targeting *aflR*, *aflS*, *aflC*, *pes1*, and *aflep* in peanut [108,109]. In a RIL population derived from the cross between Xuhua13 (susceptible) and Zhonghua 6 (resistant), RNA-seq analysis led to the identification of *AhAftr1*, an NB-LRR-encoding gene featuring a unique structural variation in its LRR domain [95]. Functional studies validated *AhAftr1* as a key player in aflatoxin resistance via the ETI pathway. Beyond peanuts, similar strategies have shown efficacy in maize. Targeted silencing of *aflR*, *aflM*, and *aflC*, along with developmental genes such as *alk*, *amy1*, and *p2c*, has led to reduced *A. flavus* growth and aflatoxin content in transgenic maize lines [39,110,111,112,113,114], demonstrating cross-crop potential for these gene-targeting strategies.

### 5.2. Overexpression of Defensins, Chitinases, and PR Proteins in Host

Parallel to silencing strategies, overexpressing host defense genes has provided another layer of protection. When expressed in peanuts, the rice-derived chitinase gene RChit enhanced resistance to *A. flavus*, offering an effective defense mechanism by degrading fungal cell walls [84]. Similarly, transgenic peanut lines overexpressing the *ARAhPR10* gene, belonging to the PR10 family, showed reduced fungal colonization and lower aflatoxin content [115]. Sundaresha et al. (2010) demonstrated that introducing a tobacco β-1,3-glucanase gene further bolstered peanut defenses against fungal invasion [116]. Recent studies have identified *AhS5H1* and *AhS5H2* in peanut as salicylate 5-hydroxylases that convert SA into 2,5-dihydroxybenzoic acid (2,5-DHBA) [101]. Overexpression of these genes in *Arabidopsis* and *Nicotiana benthamiana* resulted in significantly reduced SA levels and enhanced susceptibility to *Pseudomonas syringae* pv. tomato DC3000 [101]. Conversely, maintaining higher SA levels by suppressing *AhS5H* expression correlates with improved resistance. These findings highlight *AhS5H1* and *AhS5H2* as susceptibility factors, where overexpression compromises SA-dependent defense and enhances vulnerability to SA-sensitive pathogens like *P. syringae* [101]. These genetic engineering successes demonstrate that both disabling fungal virulence factors and enhancing host defense genes can significantly reduce aflatoxin. Another complementary approach is to alter the host’s own S genes to create inherent resistance, which we discuss next. Numerous gene targets have been explored for transgenic and gene-silencing approaches to mitigate *A. flavus* infection and aflatoxin contamination. Table 1 summarizes the major host and fungal gene targets, associated outcomes, and experimental interventions that have been validated in peanut.

## 6. Exploiting Plant Susceptibility Genes (S) for Durable Resistance

Plant susceptibility (S) genes are native host genes that are co-opted by pathogens to facilitate infection, colonization, or toxin production [118]. Emerging evidence suggests that *A. flavus* relies on a subset of these host genes to weaken defense responses and promote aflatoxin biosynthesis. Unlike resistance (R) genes, which trigger immune responses, S genes act as vulnerability points in the plant’s defense network [119]. Disrupting or silencing these genes can lead to recessively inherited, durable resistance without the need to introduce external resistance alleles [120,121,122,123,124].

### 6.1. Identification and Functional Validation of S Genes

Traditional breeding has largely focused on dominant R genes, which recognize pathogen effectors and trigger defense responses. However, these resistances are often short-lived due to the high mutation rate of pathogen effectors, allowing them to evade host recognition. In response, gene stacking, introducing multiple R genes into one genotype, has emerged as a strategy to broaden and stabilize resistance [125]. Yet, a parallel and increasingly compelling approach targets S genes that pathogens exploit for successful colonization. Disrupting or silencing these genes can result in recessive, durable, and occasionally broad-spectrum resistance [102,118,121].

The discovery of S genes typically follows either forward or reverse genetics approaches. Forward genetics screens for mutants with altered susceptibility and has traditionally relied on model organisms like *Arabidopsis thaliana* [126]. Reverse genetics, by contrast, begins with candidate genes identified via gene expression profiling or pathogen effector studies and validates them using RNAi or HIGS techniques [121]. Another powerful method involves identifying host proteins targeted by pathogen effectors, often using yeast two-hybrid assays to detect key interactions [127]. Furthermore, the evolutionary conservation of S genes enables ortholog mining in crops. Comparative phylogenetic analyses, supported by transcriptome and genome sequencing, allow prediction and functional exploration of candidate S genes [126,128]. In peanuts, identifying and editing S genes represents a transformative strategy for mitigating aflatoxin contamination. Candidate genes such as *MLO*, *AhS5H1*, *AhS5H2* (salicylic acid 5-hydroxylase genes), and *ABR1* (an ABA-responsive transcription factor) have emerged as key players involved in host susceptibility to *A. flavus* infection and aflatoxin biosynthesis [100,101,102]. These genes are frequently associated with immune suppression or hormonal signaling pathways that pathogens exploit. In recent studies, these candidate genes have been proposed for functional validation based on consistent differential expression patterns between resistant and susceptible genotypes, functional annotations implicating them in hormone-regulated immunity, and sequence homology to characterized S genes in model plants such as *Arabidopsis thaliana* [100,101,102,121,126].

The S genes are functionally diverse and fall into three general categories [124]. The first category includes genes facilitating pathogen entry, such as the *MLO* (Mildew resistance locus O) gene family, whose inactivation provides strong resistance to powdery mildew in several crops [118,129]. The second comprises negative regulators of plant immunity, such as *DMR6* in *Arabidopsis thaliana*, whose loss results in elevated salicylic acid (SA) levels and resistance to *Hyaloperonospora parasitica* [130]. The third category includes compatibility genes co-opted by pathogens for nutrition and proliferation, exemplified by rice’s SWEET sugar transporter family. *OsSWEET11* is transcriptionally activated by TALEs from *Xanthomonas oryzae* pv. *oryzae*, promoting sugar efflux to nourish the pathogen [131,132]. Loss-of-function mutations in SWEET genes have been shown to confer broad-spectrum resistance against *Xanthomonas* in multiple crops. In rice, targeted editing of *OsSWEET13* and *OsSWEET14* disrupted TAL effector-mediated activation, providing resistance to various *Xanthomonas oryzae* strains and reducing susceptibility to brown planthopper [117,133]. Similar resistance phenotypes have been observed in cassava [134], cotton [135], and citrus, where CRISPR/Cas9-mediated knockout of *CsSWEET15* significantly reduced disease symptoms caused by citrus canker and huanglongbing [120]. Disabling S genes can restrict pathogen survival, while comparative genomics enables the identification of conserved S gene homologs across plant species [118,121]. As our understanding of S gene functions deepens, targeting these genes offers a promising strategy for crop breeding to improve disease resistance. Table 2 provides a summary of identified and candidate S genes in peanut, their functional roles, and their potential as genome editing targets for durable resistance.

### 6.2. Genome Editing and Precision Breeding Tools for S Gene Manipulation

New precision breeding technologies, including genome editing platforms like ZFNs, TALENs, and especially CRISPR/Cas9, have revolutionized the efficient manipulation of S genes, offering high specificity with minimal off-target effects [136,137,138,139]. Among these, CRISPR/Cas9 has gained prominence for its simplicity, specificity, and versatility, enabling targeted gene knockouts, insertions, and fine-scale modulation of gene activity. Phogat et al. (2024) recently provided a comprehensive review detailing notable progress in peanut transformation efficiency, delivery technologies, and genome editing approaches [140]. Beyond the ubiquitous Cas9, alternative CRISPR systems like Cas12a (formerly Cpf1) are broadening the plant genome editing toolkit. Cas12a recognizes a distinct thymine-rich PAM sequence (5′-TTTV), allowing access to genomic regions less amenable to Cas9 targeting [141]. It also relies on a shorter CRISPR RNA and possesses intrinsic RNase activity for processing CRISPR arrays, enabling efficient multiplex editing from a single transcript. Importantly, Cas12a induces DNA breaks with staggered ends that often result in larger deletions, an advantage for knocking out susceptibility genes [141]. While not yet demonstrated in peanut, Cas12a represents a promising genome editing tool due to its high editing efficiency, T-rich PAM recognition, and multiplexing capability. It has been successfully applied in several crops such as rice, maize, and cotton, and thus holds potential as a future strategy for legume editing [142]. However, its efficacy in peanut remains to be experimentally validated. The expanding CRISPR nuclease repertoire provides breeders with greater flexibility in choosing optimal tools for specific targets and contexts.

Prime editing represents another leap in genome-editing technology, offering a versatile “search and replace” capability for targeted DNA modifications [143]. Prime editing employs a fusion of Cas9 nickase with a reverse transcriptase, guided by a specialized prime editing gRNA (pegRNA) that encodes the desired edit, to directly install small insertions, deletions, or base substitutions at the target site. Although still nascent in plant systems, prime editing has been successfully implemented in several crops (including rice, wheat, and tomato) and is being optimized for higher efficiency [144,145]. In legumes like peanut, initial experiments have shown promise. Biswas et al. (2022) reported the use of prime editing in peanut protoplasts to correct a mutant GFP reporter gene, achieving detectable but low editing efficiencies (~0.2–0.5%) [146]. These early results highlight both the potential and the current technical challenges of prime editing in peanut. The continued refinement of base and prime editors is poised to complement CRISPR/Cas9 and Cas12a, ultimately providing a spectrum of tools for fine-tuning the peanut genome for aflatoxin resistance. These precision tools offer breeders a robust toolkit to develop resistant crops by neutralizing pathogen susceptibility factors.

One of the most powerful aspects of genome editing lies in its capacity for multiplexing simultaneous editing of multiple loci within a single generation. This capability facilitates precision trait stacking and pyramiding of resistance alleles. For instance, concurrent disruption of several S genes or resistance-associated QTLs can lead to additive or synergistic effects, enhancing durability and breadth of resistance under diverse environmental conditions [112]. Various multiplexing strategies have been developed, such as polycistronic tRNA-sgRNA expression cassettes and exploitation of Cas12a’s ability to process CRISPR arrays, enabling simultaneous targeting of numerous genomic sites [141]. This allows researchers to pyramid multiple resistance mechanisms in a single genetic background, an approach that could significantly strengthen aflatoxin resistance in peanut. CRISPR/Cas9-based allele pyramiding can also be integrated with other technologies, such as RNAi and HIGS, enabling multi-tiered defense strategies targeting fungal infection and toxin production. The combination of these advanced molecular tools holds significant promise for developing peanut cultivars with broad-spectrum and long-lasting resistance to *A. flavus* and aflatoxin contamination.

Despite their promise, both CRISPR/Cas12a and prime editing face significant technical challenges in peanut. Cas12a has not yet been demonstrated in peanut, and its deployment is further hampered by transformation and regeneration inefficiencies across genotypes. These issues include the lack of robust tissue culture protocols optimized for Cas12a, as well as potential targeting limitations due to its T-rich PAM requirement (5′-TTTV). Broader application may require codon optimization and the development of PAM-relaxed Cas12a variants [147]. Prime editing has only been tested in peanut protoplasts, where a study reported the correction of a mutant GFP reporter gene, but at very low efficiencies (~0.2–0.5%) [148]. No prime edits have been stably transmitted to regenerated plants. These limitations stem from challenges in pegRNA design, delivery efficiency, and ineffective DNA repair pathways in transformed cells. Recent reviews of peanut genome editing technology have highlighted tissue culture-free delivery strategies including nanoparticle-mediated gene transfer, viral vectors, pollen magnetofection, pollen tube injection, node injection, and vacuum infiltration as promising avenues for overcoming genotype-dependent transformation and regeneration challenges in peanut [149].

### 6.3. Pleiotropic Effects and Trade-Offs

Despite their benefits, impairing S genes can sometimes cause undesirable pleiotropic effects. Since many S genes also serve critical roles in plant growth or development, their inactivation may lead to side effects such as dwarfism, early senescence, or reduced abiotic stress tolerance [126]. For instance, in barley, *HvMLO* mutants show premature leaf senescence under certain conditions [118]. Similarly, *DND1* silencing in tomatoes caused severe growth defects, while in potatoes, its effects were mild and environmentally dependent [150]. In citrus, mutation of *CsSWEET15* led to mild chlorosis and stunted growth over time, particularly in older plants and when combined with other SWEET gene mutations, likely due to impaired phloem loading and reduced sugar transport [120]. Nonetheless, careful allele selection and background breeding can mitigate these effects. In many cases, mild loss-of-function variants or context-specific editing offer resistance without compromising plant fitness. As understanding of gene function and regulatory networks deepens, the targeted use of S genes remains a viable and powerful avenue for durable resistance. Moreover, targeted editing of S genes using CRISPR/Cas9 can reduce susceptibility and boost resistance without transgene introduction, offering regulatory advantages in non-GMO breeding contexts. Additionally, editing of non-coding regions such as cis-regulatory motifs or promoter elements offers opportunities for fine-tuning gene expression, thereby achieving resistance while minimizing potential pleiotropic effects. Although such effects have not yet been reported in peanuts, current efforts to edit susceptibility genes remain largely at the proof-of-concept stage, and no studies have systematically evaluated the pleiotropic consequences of these edits. This remains a key consideration for future research to ensure that resistance traits do not compromise growth, yield, or abiotic stress tolerance.

### 6.4. Candidate S Genes in Peanut

Although S gene research in peanut is still emerging, several studies have identified promising candidates. Prasad et al. (2023) used HIGS to silence fungal genes (*nsdC*, *veA*, *aflM*, *aflR*) [107]. They observed the upregulation of host susceptibility-associated proteins (SAPs) like annexins, syntaxins, calmodulin, and MLO-family proteins in wild-type plants. These findings position SAPs as potential targets for gene editing in future resistance breeding efforts. In a genome-wide analysis, Traore et al. (2021) reported 25 *AhMLO* loci across 14 chromosomes, with two loci in Clade V associated with powdery mildew susceptibility in other crops highlighted as strong S gene candidates [102]. The presence of defense-related cis-elements such as TC-box and thymine-rich motifs in their promoters further supports their functional relevance [151,152]. Liang et al. (2023) identified *AhS5H1* and *AhS5H2*, two SA hydroxylase genes whose overexpression reduced endogenous SA and increased 2,5-DHBA levels, heightening susceptibility to *Pseudomonas syringae* pv. tomato DC3000 [101]. These genes likely interfere with SA-mediated defenses and are strong candidates for functional knockouts. Finally, Clevenger et al. (2016) proposed the ethylene-responsive transcription factor *ABR1* as a potential peanut S gene [100]. As a repressor of ABA signaling, *ABR1* may influence pre-harvest aflatoxin susceptibility, acting as a molecular switch during fungal infection. These candidate genes lay a foundational roadmap for future peanut breeding efforts focused on loss-of-susceptibility traits. Their functional validation and precision editing hold great promises for developing peanut varieties with stable resistance to *A. flavus* and reduced aflatoxin contamination.

While empirical evidence on peanut S genes remains limited, the candidates discussed, such as *AhS5H1/2*, *ABR1*, and *MLO*-like proteins, offer compelling starting points for functional validation. Although homologs of other known susceptibility genes from model species, such as *DMR6*, *DND1*, or *NPR1*, are likely present in peanut, their roles in aflatoxin-related susceptibility remain speculative and unverified. As no peanut S gene has yet been intentionally knocked out using CRISPR/Cas systems, future research should prioritize functional screens through targeted gene editing or TILLING of these candidate genes to assess whether their loss-of-function confers enhanced resistance without adverse agronomic effects. Such efforts would not only confirm the causal roles of these loci but also open the door to practical applications in breeding for durable, non-transgenic aflatoxin resistance. Disabling functional S genes can prevent fungal colonization and aflatoxin accumulation, while several biotechnological tools are now available to precisely target these loci.

## 7. Future Roadmap and Conclusions

Over the past two decades, significant advances have been made in understanding the complex biology of aflatoxin contamination in peanuts, alongside the development of molecular and genomic strategies aimed at mitigating its impact. Despite these achievements, the development of peanut cultivars with stable, heritable, and field-effective resistance to aflatoxin remains a critical and largely unmet objective [99]. One of the major reasons for this is the quantitative nature of aflatoxin resistance, which is typically governed by multiple small-effect loci. These loci are often environmentally responsive, with resistance expression strongly influenced by abiotic and biotic stress factors, such as drought, elevated temperatures, and insect damage. These stressors collectively create favorable conditions for *A. flavus* colonization and aflatoxin biosynthesis [72]. In addition to the complex inheritance of resistance traits, the genomic architecture of peanut poses additional challenges. Cultivated peanut is an allotetraploid species, comprising two highly similar sub genomes (A and B), which exhibit substantial gene redundancy and the presence of homeologous gene pairs [153]. This polyploidy complicates both traditional QTL mapping and modern genome-editing efforts, where distinguishing between homeolog-specific gene copies and achieving simultaneous edits across multiple gene copies becomes particularly difficult. Moreover, peanut transformation protocols remain inefficient and genotype-dependent, limiting the routine deployment of transgenic or edited lines for research or commercial purposes [140].

**Figure 3:**
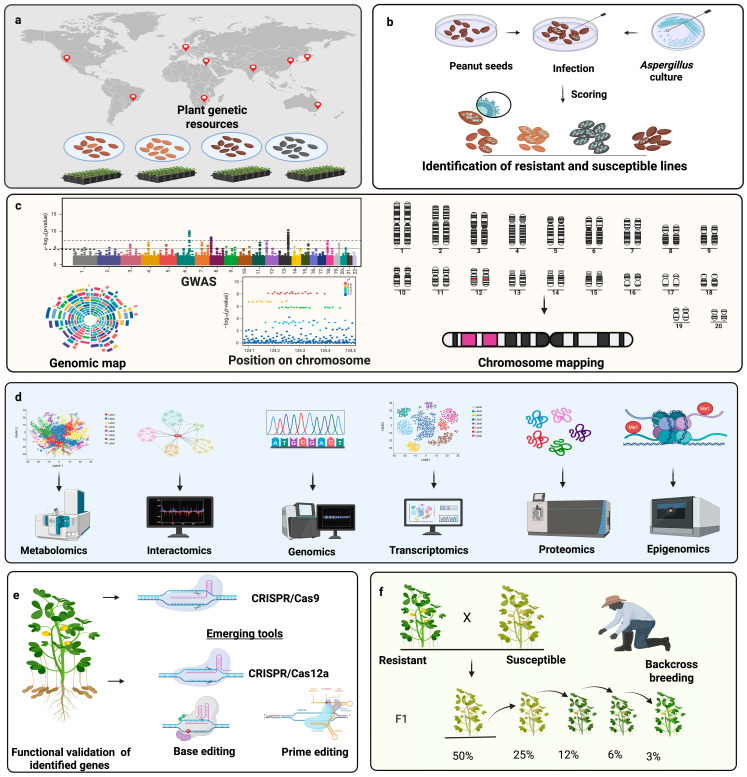
Integrated pipeline for developing aflatoxin-resistant peanut cultivars. (**a**) Strategic collection of genetically diverse germplasm, including wild relatives, landraces, and elite lines from global locations to capture natural variation in resistance traits. (**b**) Artificial inoculation of seeds with *Aspergillus flavus* under controlled conditions, followed by phenotypic screening for resistance through fungal colonization and aflatoxin quantification. (**c**) Integration of phenotypic data using genome-wide association studies (GWAS) and quantitative trait loci (QTL) mapping to identify key loci. (**d**) Deployment of multi-omics platforms, including genomics, transcriptomics, proteomics, metabolomics, epigenomics, and interactomics to unravel resistance mechanisms and prioritize candidate genes. (**e**) Functional validation of candidate genes using advanced genome editing technologies. While CRISPR/Cas9 has been successfully applied in peanut, other systems such as Cas12a, base editors, and prime editors are considered promising tools but remain to be experimentally validated in this crop. (**f**) Introgression of validated resistance alleles or edited loci into elite cultivars using marker-assisted backcrossing and genomic selection to develop high-performing, aflatoxin-resistant lines.

To overcome these obstacles, an integrated pipeline is essential (Figure 3). The pipeline illustrates the need to integrate phenotyping, genetic mapping, omics technologies, gene editing, and precision breeding into a unified framework. The first step involves the strategic collection of genetically diverse peanut germplasm from global repositories and agroecological zones. Broadening the genetic base is critical for capturing naturally occurring variation in resistance traits, especially in wild relatives and landraces that may harbor valuable alleles absent in elite cultivars. Next, these accessions are subjected to high-throughput phenotyping under both controlled and stress-inducing field conditions. Artificial inoculation assays, including in vitro seed colonization and pre-harvest aflatoxin quantification, enable accurate classification of resistant and susceptible genotypes [99]. Once reliable phenotypic data are available, the next phase involves integrating these data with genetic mapping strategies. QTL mapping in biparental populations and GWAS in diverse panels allow researchers to identify genomic regions associated with resistance traits [90,91,93]. These analyses provide a foundation for marker development, which can be used for marker-assisted selection (MAS) and to anchor multi-omics investigations. Importantly, combining QTLs and GWAS results with meta-analysis and haplotype-based approaches enhances the identification of stable, environment-independent loci across diverse genetic backgrounds [154]. Identification of the key loci opens the door to a deeper understanding of underlying biological mechanisms through multi-omics approaches. Transcriptomics reveal differentially expressed genes during pathogen infection; proteomics uncovers post-translational regulation and protein interactions; metabolomics highlights resistance-associated biochemical signatures; epigenomics explores methylation and chromatin changes; and interactomics identify regulatory and protein–protein interaction networks. Integration of these omics layers provides a systems-level view of the peanut *A. flavus* interaction, enabling the prioritization of high-confidence candidate genes within mapped loci [155]. Functional validation of these candidates is the next crucial step. Genome editing tools such as CRISPR/Cas9, Cas12a (Cpf1), base editors, and prime editing systems offer precise and versatile platforms for dissecting gene functions in peanut. These technologies allow researchers to knock out susceptibility genes, introduce resistance alleles, or correct detrimental variants with minimal genomic disruption. Multiplex genome editing holds special promise in peanut, where edits in multiple homeologous gene copies may be required to observe a phenotypic effect. Despite the promise, off-target effects, efficiency of editing in polyploid contexts, and the regeneration of edited plants remain areas for further optimization [156]. Following validation, elite cultivars can be improved by introgressing beneficial alleles or edited loci through backcross breeding combined with MAS or genomic selection. Moreover, pyramiding of multiple resistance QTLs alongside susceptibility gene knockouts may offer broad-spectrum, durable resistance that is less likely to be overcome by pathogen adaptation. Validation of improved lines under greenhouse, confined field trials, and multi-location environments is essential to confirm trait stability, yield performance, and resistance durability [157]. In the future, the integration of machine learning models, predictive breeding algorithms, and digital phenotyping platforms could further streamline this pipeline. Real-time stress monitoring, remote sensing, and UAV-based imaging can provide non-destructive assessments of aflatoxin contamination risk, supporting proactive breeding and management decisions [158].

In conclusion, the development of aflatoxin-resistant peanut cultivars requires a multidisciplinary and data-driven approach. This integrated strategy offers a realistic path forward to deliver aflatoxin-safe peanut cultivars that meet the demands of growers, processors, and consumers in both developed and developing regions.

## Figures and Tables

**Table 1:** Host-induced gene silencing, RNA interference and overexpression studies in peanuts.

Method	Target Gene (s)	Outcome
HIGS	*aflM*, *aflP*	Inhibit aflatoxin production [106]
Overexpression	*MsDef1*, *MtDef4*.2	Reduced *A. flavus* infection and aflatoxin production [106]
HIGS (multiplexed)	nsdC, veA, aflM, aflR	Lowered infection and aflatoxin accumulation [107]
RNAi	*aflR*, *aflS*, *aflC*, *pes1*, *aflep*	Lowered aflatoxin accumulation [108,109]
Overexpression	*RChit* (rice chitinase)	Enhanced resistance [84]
Overexpression	ARAhPR10	Reduced *A. flavus* infection [115]
Overexpression	*β-1,3-glucanase* (tobacco)	Resistance to *A. flavus* [116]
Natural variant	*AhAftr1*	Resistance via ETI [117]
Overexpression (S-gene validation)	*AhS5H1*/2	Increased susceptibility (SA depletion) [101]
Overexpression (S-gene candidate)	*ABR1*	Potential role in susceptibility [100]
Candidate S-genes	*MLO*-like, Annexin, Syntaxin, Calmodulin-like proteins	Potential susceptibility gene targets [102,107]

**Table 2:** Candidate susceptibility genes in peanut for genome editing.

Candidate S-Gene	Functional Role	Evidence Source	Proposed Intervention
*AhS5H1/2*	SA hydroxylases (SA catabolism)	Overexpression increases susceptibility [101]	Knockout to restore SA accumulation
*ABR1*	ABA-responsive transcription repressor	Expression linked to susceptibility [100]	Gene knockout or downregulation
*MLO*-like	Defense suppression membrane protein	Homologs identified in peanut [102]	CRISPR knockout
Annexins/Syntaxins	Vesicle trafficking	Upregulated in infected tissues [107]	Functional knockout or silencing
Calmodulin-like	Calcium signaling modulator	Expression increased under stress [107]	Functional knockout or silencing
*DMR6*-like	SA 5-hydroxylase analog	Homologs predicted (not yet validated)	Gene knockout

## Data Availability

The original contributions presented in this study are included in the article. Further inquiries can be directed to the corresponding author.
